# 25C-NBOMe: Preliminary Data on Pharmacology, Psychoactive Effects, and Toxicity of a New Potent and Dangerous Hallucinogenic Drug

**DOI:** 10.1155/2014/734749

**Published:** 2014-07-03

**Authors:** Francesco Saverio Bersani, Ornella Corazza, Gabriella Albano, Giuseppe Valeriani, Rita Santacroce, Flaminia Bolzan Mariotti Posocco, Eduardo Cinosi, Pierluigi Simonato, Giovanni Martinotti, Giuseppe Bersani, Fabrizio Schifano

**Affiliations:** ^1^Department of Neurology and Psychiatry, Sapienza University of Rome, 00185 Rome, Italy; ^2^Department of Postgraduate Medicine, School of Life and Medical Sciences, University of Hertfordshire, AL10 9AB Hatfield, UK; ^3^Department of Medical-Surgical Sciences and Biotechnologies, Sapienza University of Rome, 04100 Latina, Italy; ^4^Department of Neuroscience and Imaging, University “G. D'Annunzio,” 66100 Chieti, Italy; ^5^International Association for Applied Human Sciences (A.I.A.S.U.), 00100 Rome, Italy

## Abstract

*Introduction*. The use of novel psychoactive substances (NPSs) has rapidly increased as well as their online availability. The aim of this paper is to provide a comprehensive review of the nature and the risks associated with 25C-NBOMe, which has recently appeared in the drug market. *Methods*. A systematic analysis of the scientific literature and a qualitative assessment of online and media resources (e.g., e-newsgroups, chat-rooms, and e-newsletters) in 10 languages were carried out. *Results*. 25C-NBOMe is sold online as legal LSD or as research chemical with different designations such as “Boom,” “Pandora,” “Holland film,” or “N-bomb.” It is a partial agonist of 5-HT2A receptors. It is usually ingested orally/sublingually and, less commonly, nasally, through injection, vaginally, rectally, and smoked. Its effects include sublingual numbing, stimulation, “body high,” hallucinations, dissociation, and anxiety. 25C-NBOMe presents high risk of overdoses; acute toxicity and fatalities have been reported. *Conclusions*. 25C-NBOMe consumption represents an emerging phenomenon with potential harmful effects. Its use is increased by its online availability at low costs. Health and other professionals should be informed about this new trend of substance use.

## 1. Introduction

In the last few years, the recreational use of novel psychoactive substances (NPS) has rapidly increased as well as their availability in the drug market. This new phenomenon represents not only an unprecedented challenge in the field of drug addiction, but also a fast growing problem from social, cultural, legal, and political perspectives [1–3].

Throughout 2013, a new group of toxic phenethylamine derivatives called NBOMe have gained prominence [4, 5]. They are phenethylamine derivatives of the 2C class of hallucinogens, initially mentioned in Alexander and Ann Shulgin's book PIHKAL, which was a contentious publication that thoroughly described the properties of a large variety of psychoactive substances [6, 7].

One of the most common substances within NBOMe group is 25C-NBOMe [4]. As a result of its recreational use, various episodes of acute intoxication and fatalities have recently been reported [4, 8, 9]. In addition, while a danger of 25C-NBOMe is its general use by those who planned to ingest it, a second danger is the accidental ingestion of the compound by individuals ingesting counterfeited LSD; according to anecdotal and media reports and scientific testing, in fact, LSD users may often unwittingly ingest the more dangerous 25C-NBOMe instead of LSD [10].

Scientific evidence on pharmacology and effects of 25C-NBOMe are poor. Through a systematic analysis of medical-scientific literature and online resources (e.g., websites, drug fora, and chat-rooms), the aim of the present paper was to provide an initial comprehensive review of the pharmacology, metabolism, toxicity, and psychoactive effects of the 25C-NBOMe compound, namely, 2-(4-chloro-2,5-dimethoxyphenyl)-N-[(2-methoxyphenyl)methyl]ethanamine.

## 2. Methods

A literature search on 25C-NBOMe was carried out in PsycINFO, Scopus, and PubMed databases. Results were integrated with a multilingual qualitative assessment of a range of websites, drug fora, and other online resources (i.e., e-newsgroups, chat-rooms, mailing lists, e-newsletters, and bulletin boards); between January 2013 and January 2014 exploratory qualitative searches of 153 websites in English and Italian took place using generic and specific keywords, such as “legal highs,” “research chemicals,” “online pharmacy,” “25C-NBOMe,” “NBOMe,” “hallucinogens,” “hallucinogenic substances,” and “online pharmacies” in Google search engine. Of these, 43 were considered to be relevant for the study and as such were monitored on a regular daily, weekly, or monthly basis, depending on their relevance. The remaining 110 websites were considered not to bear any interest for the present study and thus were no longer monitored.

Further specific searches in the database provided by the Global Public Health Intelligence Network (GPHIN) also took place. This is a secure Internet-based early warning system that gathers preliminary reports of public health significance by monitoring global media sources near “real time,” 24 hours a day, 7 days a week basis. GPHIN is operated by the Public Health Agency of Canada and monitors news sources and websites across the globe in 9 languages (e.g., English, French, Farsi, Portuguese, Arabic, Russian, Spanish, and Chinese simplified/traditional) [11]. While a series of algorithms were used and adjusted to capture relevant information, analysis of relevant data since 2003 was also carried out manually by a multidisciplinary and multilingual team of analysts.

Permission for the study was granted by the School of Pharmacy Ethics Committee, University of Hertfordshire, Hatfield, UK (November 2013; PHAEC/10-42).

## 3. Results

### 3.1. 25C-NBOMe: An Emerging Hallucinogenic Substance in the Drug Market

25C-NBOMe, also known as NBOMe-2CC, Boom, C-Boom, Cimbi-82, Pandora, N-bomb, Holland film, and Dime [12], has been first synthesized in 2009 and first mentioned in the scientific literature in 2011 by Ettrup et al. [13, 14]. To our knowledge, the first case of 25C-NBOMe ingestion was recordedin 2010 [15]. The drug is believed to be manufactured in China, but shipments from Europe and Canada have also been reported [5].

**Figure 1:**
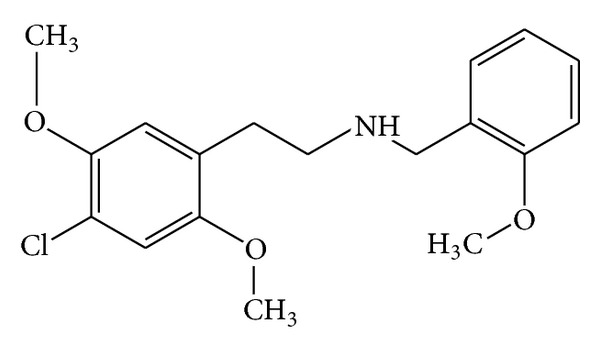
Chemical structure of 25C-NBOMe.

25C-NBOMe is a N-(2-methoxy)benzyl derivative of the psychedelic phenethylamine 2C-C (4-chloro-2,5-dimethoxyphenethylamine) [16] (Figure 1). It is derived from 2C-C by substitution on the amine nitrogen with a 2-methoxybenzyl (BOMe) group. 2C-C belongs to a group of modified phenethylamine structures called dimethoxyphenyl-ethanamines, also known as 2C substitutes [16]. The terminology “2C” was created by Alexander Shulgin et al. to describe the two carbons between the amino group and the benzene ring in the chemical structure [6, 16]. The 2-methoxybenzyl group attached at a nitrogen atom in 25C-NBOMe acts as protecting group in order to preserve the functionality of the amine group during a reaction [12, 16]. This structural change allows a 16-fold increase in potency as compared to other NBOMe compounds [16].

### 3.2. Pharmacology

The pharmacological properties of NBOMe series were first investigated by Heim and collaborators [17]. 25C-NBOMe acts as a potent partial agonist for the 5-HT2A receptor and has been studied in its 11C radiolabelled form as a potential ligand for mapping the distribution of 5-HT2A receptors in the brain using positron emission tomography (PET) [13].

25C-NBOMe is characterized by nanomolar affinity towards the 5-HT2A receptor and has an agonistic binding affinity of 2.89 ± 1.05 nM in vitro [13]; consistently, it has been described by Braden et al. as “superpotent” agonist of the 5-HT2A receptors [18] and it is pharmacologically active even at very low submilligram doses [16].

### 3.3. Availability

25C-NBOMe has entered the drug market in 2010 in the forms of blotter papers and liquid and less commonly as tabs [12, 16]. Since then its diffusion has been facilitated by an increased online availability on websites where it is sold as “a legal alternative of lysergic acid diethylamide (LSD)” or as a research chemical “not for human consumption,” as well as in combination with other substances such as 25D-NBOMe or 2,5-dimethoxy-4-iodoamphetamine (DOI) [12]. Its price ranges from about $7 to about $10 [19].

### 3.4. Route of Administration and Dosage

25C-NBOMe can be consumed through several routes of administration. The most common route of administration is the oral or sublingual ingestion (mixed with solvents such as alcohol) by soaking the liquid on a blotter and keeping it on the buccal mucosa for several minutes or swallowing it; Lawn et al. undertook a survey study through the Global Drugs Survey reporting that 81.2% of users administered the drug orally or sublingually [20]. Less commonly, 25C-NBOMe can be taken nasally (insufflation and absorption of liquid solutions), through injection (intravenously and intramuscularly), vaginally, and rectally, and it can be smoked as freebase [12, 20, 21].

Several independent reports suggest that doses of swallowed 25C-NBOMe range between 50 and 1200 *μ*g and that hallucinogenic effects can be achieved at a dose of 50–200 *μ*g [12, 15, 21]. When administered sublingually, the threshold for the onset of hallucinogenic effects reportedly is about 100–250 *μ*g, with mild effects after 250–450 *μ*g, strong after 450–800 *μ*g, and very strong over 800 *μ*g [12]. The effects of insufflated 25C-NBOMe have been described as light after 50–200 *μ*g, mild after 200–350 *μ*g, strong after 350–700 *μ*g, and very strong after higher doses [12]. Finally, several independent users report to smoke the substance dissolving 10 mg of 25C-NBOMe freebase in 30 mL 99.9% isopropanol and then to dry 1 mL “doses” (300 *μ*g) onto plant matter to be smoked in cigarettes or pipes; in this latter case, the threshold for the onset of intense hallucinogenic effects is about 50–200 *μ*g [22].

Example doses reported on the drug fora involving more rare routes of administration include “830 *μ*g, injected,” “400 *μ*g, rectally,” and “500 *μ*g, vaginally” [21, 23, 24].

### 3.5. Effects

*(i) Sublingual Numbness.* When taken sublingually, the first effects are anecdotally described as unpleasant. These include a metallic chemical taste along with a sense of numbness of the tongue and mouth, which can last up to an hour after the ingestion. Numbness of the tongue and mouth has been reported to be a key difference between blotter papers containing LSD and those containing NBOMe drugs [25].

*(ii) Body High.* The “body high” can be described as a generally mild, all-encompassing, soft but euphoric tingling sensation. This tingling sensation is also accompanied by spontaneous rushes of euphoria which become longer and more drawn out proportional to the dosage consumed [12, 25].

*(iii) Stimulation.* 25C-NBOMe can have stimulants effects. According to subjective reports, it generates a “unique” sense of stimulation, which has been described as “energetic” but at the same time devoid of any physical movement, unless intentional. For some users, the stimulation can be quite uncontrollable, occasionally resulting in bodily shakes and a grinding of the teeth comparable to that of MDMA and traditional stimulants such as amphetamine [12, 25].

*(iv) Psychedelic Effects.* They can differ significantly. These include introspection, euphoria, acceleration of thought, conceptual thinking, time distortion, increased empathy, and sociability. Other effects, which become more common as dosages increase, include depersonalization, derealization, anxiety, dissociation, panic, and fear [25]. In addition, 25C-NBOMe is able to cause a wide range of hallucinatory states, including visual and auditory hallucinations [12, 25]. Visual effects of the drug include increased visual acuity, enhanced pattern recognition, enhanced colors, and distortions (e.g., visual drifting, texture repetition, and tracers) [12, 25]. A summary of 25C-NBOMe effects is given in Table 1.

A user reported the effects of a 500 *μ*g 25C-NBOMe nasal insufflation as follows:

*The kitchen started to swirl around, everything became very colourful, the intensity increased exponentially. It became way way more intense than I had expected very very quickly. Panic started to take hold and no matter what I did I could not shake it off. I tried to reassure myself and tried to calm down but as my world started to become more and more chaotic and as I started to completely lose myself I found this impossible to do… Then things started to get really really nasty. The thoughts in which the loop seemed to be wrecking peoples' lives were interlaced with the thoughts that somehow I was doing something so terrible, so humiliating and disgusting that the whole world thought I was a joke and that I did not deserve to live. I was completely dissociated and out of the room, I was on the ground outside being pelted with rubbish by hundreds of people. There were ambulances, police cars and my dad all whirling round. This image remained for a long time. However after a while it was as if people understood and whilst some still hated me others were rooting me on to make it through to the other side of this trip. [12]*

Onset and duration of the effects largely depend on the doses and the routes of administration (more details are given in Table 2) [12, 15, 21]. Overall, the insufflation of 25C-NBOMe seems to lead to more rapid, severe, and toxic consequences than the oral/sublingual ingestion. Residual long-lasting symptoms have been reported even several months after 25C-NBOMe consumption [26].

### 3.6. Toxicity

Common negative physical side effects of 25C-NBOMe include vasoconstriction, nausea, vomiting, headache, irregular heartbeat, sweating, and temporary dysuria [4, 25, 27]; some users anecdotally reported to have experienced “*something terrifying to the body*” after its consumption [26]. Overdoses lead to most severe effects; some recently reported cases of intoxication were associated with confusion, agitation, hypertension, tachycardia, hyperthermia, dilated pupils, heart failure, metabolic acidosis, generalized seizure, loss of consciousness, low oxygen saturation, acute kidney, and lung failures [25, 27–29].

As 25C-NBOMe is a potent serotonergic agonist, these toxic effects may represent the clinical manifestations of serotonin toxidrome, which is known to potentially produce acute toxicity involving metabolic acidosis, rhabdomyolysis, seizures, renal failure, and disseminated intravascular coagulation.

25C-NBOMe is strongly active at extremely small doses (it exerts its effects even at microgramic levels) and users may not have precise weighing scale; therefore, the possibility of accidental overdoses is not irrelevant. In addition, the lack of knowledge of an experimentally recognized median lethal dosage (LD50), the frequent poor or mistaken dilution, and the improper handlings especially by teenagers (including the concomitant use with other drugs like methoxetamine, *α*-methyltryptamine, or synthetic cannabinoids) contribute to increase the risk of accidental overdoses of the substance and related deaths [28, 29].

Since June 2012, more than 10 fatalities have been reported as a result of the ingestion of substances in the NBOMe class [4, 27, 30–32] and at least 2 of these were attributable to 25C-NBOMe; in 2013, a 16-year-old and an 18-year-old males died in USA and UK after having inhaled the drug [9, 21, 25].

### 3.7. Legal Status

From a legislative point of view, NPSs are often identified as temporary class drugs (TCDs); the TCD is a relatively new status for controlled drugs which has been adopted in some jurisdictions, notably New Zealand, USA, and the United Kingdom, to attempt to bring newly synthesized designer drugs under legal control [33].

In the United States the Controlled Substance Act (CSA) is the federal drug policy under which the manufacture, importation, possession, use, and distribution of certain substances are regulated. CSA created five schedules (classifications), with varying qualifications for a substance to be included in each; two federal agencies, the Drug Enforcement Administration (DEA) and the Food and Drug Administration (FDA), determine which substances are added to or removed from the various schedules. Classification decisions are required to be made on criteria including potential for abuse, currently accepted medical use in treatment in the United States, and international treaties. On November 2013 the DEA added 25I-, 25B-, and 25C-NBOMe to Schedule I making these NBOMe compounds “temporarily” controlled for two years. Sale, possession, manufacture, and distribution of these three compounds are crimes under the US federal law [21, 34].

For what concerns the United Kingdom, a total of 10 benzofuran and indole analogues and four NBOMe hallucinogens (25C-, 25B-, 25I-, and 25D-NBOMe) have been classified as TCDs from June 2013 to be reviewed in June 2014. This means that sale and import of the named substances are considered criminal offences and are treated as Class B drugs [21, 35, 36].

In New Zealand, 25C-NBOMe is considered to be substantially similar in chemical structure to the illegal hallucinogen dimethoxybromoamphetamine (DOB), being therefore considered a Class C controlled drug analogue [21].

In addition, 25C-NBOMe is currently controlled under drug control legislation in Denmark, Hungary, Israel, Lithuania, New Zealand, Portugal, Romania, Russia (the first country that officially banned the compounds of NBOME series in 2011), Slovenia, Sweden, and areas of Australia (Queensland and New South Wales) [21].

## 4. Discussion

Differently from the prevalence rates of the use of internationally controlled drugs which seem generally to have stabilized in recent years, the market of NPS, also known as “designer drugs,” “herbal highs,” “synthetic drugs,” “research chemicals,” and “legal highs,” has significantly grown over the past decade [37–40]. An ever-increasing number of NPS are emerging worldwide, as witnessed through the EU Early Warning System of the European Monitoring Centre for Drugs and Drug Addiction (EMCDDA): 73 new psychoactive substances were officially notified for the first time in 2012, up from 49 in 2011, 41 in 2010, and 24 in 2009 [41]. This can be attributed to developments in manufacture, distribution, administration, and communication of new recreational substances [3, 42–44]. Therefore, it is crucial for both consumers of recreational drugs and health professionals to be aware of the effects and toxicity of NPSs so that proper toxicological investigations and eventual medical interventions can be performed.

Scientific literature on pharmacology and effects of 25C-NBOMe is limited. Through a systematic analysis of the current literature and other online resources (e.g., websites, drug fora, and chat-rooms), the present paper aimed to provide fresh insights into the properties, effects, and potential toxicity of this emerging psychoactive drug.

The lack of awareness on its potential risks and unclear legal status of phenethylamine-class drug in various countries may favor the diffusion of 25C-NBOMe among drug users. According to anecdotal and media reports and scientific testing [10], it is likely that LSD users may unwittingly ingest the more dangerous and possibly lethal 25C-NBOMe instead of LSD. On March 2013, the case of a woman who died after having assumed two blotters of what she wrongly believed to be LSD but that actually contained one of the NBOMe compounds was reported in the media [45]. As a result, emergency medical personnel should be alerted and prepared to treat patients also for the accidental ingestion of a drug in the NBOMe group, which necessitates more intensive care than would otherwise be assumed [4, 46].

The patients may require benzodiazepines and other drugs for the control of overdose-related psychiatric symptoms such as agitation, aggression, or hallucinations [28]. At the same time, all the potential organic sequelae such as hypertension, hyperthermia, heart failure, metabolic acidosis, generalized seizure, acute kidney and lung failures, and manifestations of serotonin toxidrome should be carefully monitored. Appropriate treatments might include fluids, aggressive cooling, pharmacological interventions, and other high-level resuscitative measures [4]. For example, Grautoff and Kähler required mechanical ventilation and hemofiltration to treat a 19-year-old man who had inhaled 2 mg of 25C-NBOMe; the patient recovered after 13 days in the intensive care unit [29].

A similar challenge emerged in the last few years with the diffusion of the highly toxic and long lasting (up to 3 days) hallucinogenic drug Bromo-Dragonfly, which caused a number of fatalities among LSD users before it was recognized [47]. Both 25C-NBOMe and Bromo-Dragonfly have not been approved for human consumption and can be associated with unknown side effects/adverse reactions and long term consequences [47].

From a psychiatric point of view, there is a lack of data about long-lasting mental effects of 25C-NBOMe; however, residual symptoms have been reported even several months after its consumption [26]. Research suggests that the use of psychedelic drugs in unsupervised, unscreened, and unorganized settings (e.g., what is common for recreational drug use) may result in adverse reactions such as anxiety, depression, or psychosis [48]. Given that the NBOMe compounds have never been tested in humans, their use could result in severe adverse psychiatric consequences.

The 25C-NBOMe incompletely characterized profile and the lack of routine toxicological screening tests in many laboratories make the substance hardly identifiable. Large studies are needed to systematically investigate effects, toxicity, and potential fatal role of 25C-NBOMe. Meanwhile, the attention and clinical suspicion of families and healthcare professionals towards this new substance is warranted.

One could wonder about the limitations of carrying out an assessment of substance use whilst taking into account online and media reports; in fact, it may be inappropriate to trust information obtained from the Internet without independent verification. However, the Internet plays a central role in the NPS business and therefore, given the limited amount of relevant peer-reviewed data, this seems to be the only method to obtain preliminary information about new and emergent phenomena [49, 50].

## Figures and Tables

**Table 1:** Summary of 25C-NBOMe effects.

Sublingual numbness	Metallic chemical taste Sense of numbness of the tongue and mouth

Body high	Body tingling sensation Rushes of euphoria

Stimulation	Physical energetic stimulation Bodily shakes and a grinding of the teeth

Psychedelic effects	Introspection, euphoria, acceleration of thought, conceptual thinking, time distortion, increased empathy, and sociability

**Table 2:** Onset and duration of 25C-NBOMe effects in relation to the routes of administration.

	Oral/sublingual	Insufflation
Total duration	4–10 hours	3–8 hours
Onset	0–15 minutes	0–5 minutes
Coming up	30–90 minutes	15–30 minutes
Coming down	1–4 hours	1–3 hours
